# Patient-Related Prognostic Factors for Anastomotic Leakage, Major Complications, and Short-Term Mortality Following Esophagectomy for Cancer: A Systematic Review and Meta-Analyses

**DOI:** 10.1245/s10434-021-10734-3

**Published:** 2021-09-05

**Authors:** Robert T. van Kooten, Daan M. Voeten, Ewout W. Steyerberg, Henk H. Hartgrink, Mark I. van Berge Henegouwen, Richard van Hillegersberg, Rob A. E. M. Tollenaar, Michel W. J. M. Wouters

**Affiliations:** 1 Department of Surgery, Leiden University Medical Center, Leiden, the Netherlands; 2 Department of Surgery, Amsterdam UMC, Cancer Center Amsterdam, University of Amsterdam, Amsterdam, the Netherlands; 3 Department of Biomedical Data Sciences, Leiden University Medical Center, Leiden, the Netherlands; 4 Department of Surgery, University Medical Center Utrecht, Utrecht, the Netherlands; 5 Department of Surgery, Dutch Cancer Institute – Antoni van Leeuwenhoek Hospital, Amsterdam, the Netherlands

## Abstract

**Objective:**

The aim of this study is to identify preoperative patient-related prognostic factors for anastomotic leakage, mortality, and major complications in patients undergoing oncological esophagectomy.

**Background:**

Esophagectomy is a high-risk procedure with an incidence of major complications around 25% and short-term mortality around 4%.

**Methods:**

We systematically searched the Medline and Embase databases for studies investigating the associations between patient-related prognostic factors and anastomotic leakage, major postoperative complications (Clavien–Dindo ≥ IIIa), and/or 30-day/in-hospital mortality after esophagectomy for cancer.

**Results:**

Thirty-nine eligible studies identifying 37 prognostic factors were included. Cardiac comorbidity was associated with anastomotic leakage, major complications, and mortality. Male sex and diabetes were prognostic factors for anastomotic leakage and major complications. Additionally, American Society of Anesthesiologists (ASA) score > III and renal disease were associated with anastomotic leakage and mortality. Pulmonary comorbidity, vascular comorbidity, hypertension, and adenocarcinoma tumor histology were identified as prognostic factors for anastomotic leakage. Age > 70 years, habitual alcohol usage, and body mass index (BMI) 18.5–25 kg/m^2^ were associated with increased risk for mortality.

**Conclusions:**

Various patient-related prognostic factors are associated with anastomotic leakage, major postoperative complications, and postoperative mortality following oncological esophagectomy. This knowledge may define case-mix adjustment models used in benchmarking or auditing and may assist in selection of patients eligible for surgery or tailored perioperative care.

**Supplementary Information:**

The online version contains supplementary material available at 10.1245/s10434-021-10734-3.

Esophageal carcinoma is the seventh most common and sixth most lethal malignancy worldwide.^1^ Its incidence is rising rapidly in the Western world, which might be a result of the obesity epidemic and the associated higher prevalence of gastroesophageal reflux disease. Currently, the 5-year survival rate of curatively treated esophageal carcinoma patients approximates 40–50%.^2,3^ This curative treatment consists of neoadjuvant chemo(radio)therapy followed by surgical resection. However, esophagectomy is a highly invasive procedure associated with significant postoperative morbidity. The incidence of major postoperative complications ranges around 26–31% with failure-to-rescue rates of around 18–19%.^4,5^ Reduction of (severe) complications might reduce recovery time, length of hospital stay, readmission rates, and hospital costs, and increase long-term quality of life. In addition, recurrence-free and overall cancer-related survival are negatively affected by postoperative complications.^6,7^

The implementation of Enhanced Recovery After Surgery (ERAS) protocols reduces postoperative complication rates.^8^ Further reduction of major complications may be achieved by tailormade perioperative care using personalized prehabilitation programs. In addition, benchmarking surgical outcomes in national clinical audits might lead to a further decrease of surgical morbidity.^9,10^ An audit measures quality of care using structure, process, and outcome indicators and feeds benchmarked results back to clinicians.^11,12^ Reduction of hospital variation may enhance outcomes at population level.^13^ In auditing, knowledge on patient-related prognostic factors predicting adverse outcomes is essential to establish case-mix models enabling fair hospital comparison.

We aimed to identify patient-related prognostic factors for major postoperative complications (Clavien–Dindo ≥ IIIa), anastomotic leakage, and 30-day/in-hospital mortality after esophageal cancer surgery.^14^

## Methods

The study protocol was registered in the PROSPERO database (CRD42020204787). This systematic review and meta-analyses adhered to the Preferred Reporting Items for Systematic Reviews guidelines. The PRISMA checklist is provided in Supplementary File 1.

### Criteria for Study Eligibility

All studies including patients undergoing curative-intent esophagectomy for cancer and describing patient-related prognostic factors for (1) anastomotic leakage, (2) major postoperative complications (Clavien–Dindo ≥ IIIa), and/or (3) 30-day/in-hospital mortality were considered for inclusion. Studies including patients undergoing salvage or palliative surgery were excluded. No restrictions regarding neoadjuvant therapy or tumor stage were applied. Only retrospective or prospective cohort studies and randomized controlled trials with full-text articles published in English or Dutch were included. Case reports and case series (< 40 patients) were excluded. Studies including children (< 18 years of age) or animals were excluded. No restrictions as to study publication status were applied. In case of overlapping cohorts, the study reporting on the highest number of relevant outcome measures and/or patients was included.

### Search Method

To identify all relevant publications, the Medline and Embase electronic databases were searched systematically from inception to 19 April 2021. Search terms included controlled MeSH terms in PubMed and EMtree terms in EMBASE, as well as free-text terms. The complete search strategy is presented in Supplementary File 2. No restrictions for date of publication were applied. Reference lists of identified review articles were checked for additional relevant studies. Authors were contacted in case of full-text unavailability.

### Study Selection

**Fig. 1.**
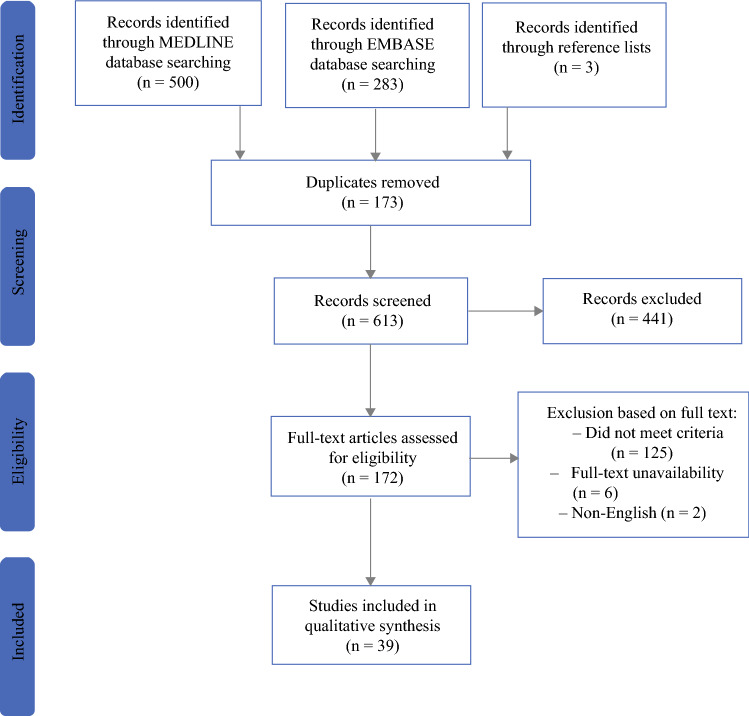
PRISMA flowchart of study selection

Study selection was performed individually by D.M.V. and R.T.v.K. Initial screening was based on title and abstract. Disagreements regarding eligibility were resolved by discussion, with M.W.J.M.W. acting as arbitrator when necessary. Thereafter, full texts were independently screened by D.M.V. and R.T.v.K. Again, M.W.J.M.W. acted as arbitrator in case of disagreement. Reasons for exclusion were documented. A flowchart of study selection is depicted in Fig. 1. Endnote X9 (Clarivate Analytics, Philadelphia, PA) and Covidence were used during the selection process.

### Assessment of Risk of Bias

All included studies were independently assessed for potential risk of bias by D.M.V. and R.T.v.K., using the Quality in Prognostic Studies (QUIPS) tool for classification of prognostic factor studies.^15^ Discrepancies were resolved by discussion, with M.W.J.M.W. as arbitrator. The risk of bias in studies was assessed in the following domains: study participation, study attrition, prognostic factor measurement, outcome measurement, adjustment bias, and statistical analysis bias. Each domain was graded as high, low, or unclear. The results are summarized in Supplementary File 3.

### Data Extraction and Management

Data extraction was performed by R.T.v.K. and subsequently checked by D.M.V. The data extraction was performed in a predefined Excel sheet, designed by D.M.V. and R.T.v.K. The sheet was piloted in at least one included study. Discrepancies regarding data extraction were resolved by discussion; M.W.J.M.W. acted as arbitrator when necessary. Subsequently, data were imputed in RevMan 5. The following data were extracted: (1) general study information (author, journal, year of publication, dataset, methodology, treatment regimen, and patient characteristics), (2) investigated patient-related prognostic factors, and (3) outcome measure incidence or odds ratios (OR) and accompanying 95% confidence intervals (CI) of outcomes in different prognostic factor groups.

### Data Analyses

Following data extraction, the homogeneity between the included studies was assessed using the Higgins *I*^2^ statistic, with *I*^2^ > 50% considered heterogeneous. Random-effect modeling was used to calculate pooled univariable OR and accompanying 95% CI for anastomotic leakage, major complications, and 30-day/in-hospital mortality. Two-sided *P * < 0.05 was considered statistically significant. Analyses were conducted using RevMan 5 (Cochrane).

## Results

After removal of duplicates, the literature search resulted in a total of 613 studies potentially eligible for inclusion. After title and abstract, and full-text screening, 39 studies met inclusion criteria (Fig. 1). The risk of bias of the included studies is depicted in Supplementary File 3. All included studies were observational. The main study characteristics are presented in Table 1. A total of 37 different patient-related prognostic factors for severe complications, anastomotic leakage, and/or 30-day/in-hospital mortality were described in 48,853 patients and used in the current meta-analyses (Table 1). Eleven studies described prognostic factors for major complications, 31 for anastomotic leakage, and 12 for 30-day/in-hospital mortality.

**Table 1:** Baseline characteristics of included studies

Author (year)	Country	Study type	Inclusion period	Number of patients	Histology (ACC/SCC)	Localization	Neoadjuvant therapy
Alexiou^16^	UK	Observational	1987–1997	523	ACC and SCC	Cervical, intrathoracic, and GEJ	0%
Aoyama^17^	Japan	Observational	2005–2018	122	ACC and SCC	Intrathoracic	–
Berkelmans^18^	The Netherlands	Observational	2013–2014	89	ACC and SCC	–	CRT 73 (82.0%) CTx 4 (4.5%)
Borggreve^19^	The Netherlands	Observational	2003–2015	406	ACC 309 (76.1%), SCC 92 (22.7%)	–	CRT 153 (37.7%) CTx 122 (30.0%)
Busweiler^20^	The Netherlands and Sweden	Observational	2012–2014	2.509	ACC 1787 (71.2%), SCC 415 (16.5%)	Cervical, intrathoracic and GEJ	CRT 1857 (74.0%) CTx 285 (11.4%)
Daele^21^	Belgium	Observational	2005–2014	412	ACC 203 (49.3%), SCC 209 (50.7%)	Intrathoracic and GEJ	RT 195 (47.3%) CTx 228 (55.3%)
Filip^22^	Italy	Observational	2008–2012	167	ACC 105 (62.9%), SCC 58 (34.7%)	Cervical, intrathoracic and GEJ	CRT 131 (78.4%)
Fjederholt^23^	Denmark	Observational	2003–2012	557	ACC 557 (100.0%)	GEJ	–
Fogh^24^	USA	Observational	1994–2005	260	–	Intrathoracic and GEJ	CRT 260 (100%)
Gao^25^	China	Observational	2016–2017	96	–	Intrathoracic and GEJ	Unspecified 38 (39.6%)
Goense^26^	The Netherlands	Observational	2012–2015	167	ACC and SCC	Intrathoracic and GEJ	CRT 8 (4.8%) CTx 145 (86.8%)
Gooszen^27^	The Netherlands	Observational	2011–2015	3.348	ACC 2600 (77.7%), SCC 663 (19.8%)	Intrathoracic and GEJ	CRT 776 (23.2%) CTx 239 (7.1%)
Hall^28^	USA	Observational	2005–2015	915	ACC 682 (74.5%), SCC 73 (8.0%)	Intrathoracic and GEJ	621 (67.8%)
Harustiak^29^	Czech Republic	Observational	2005–2012	415	ACC and SCC	–	CRT 93 (22.4%) CTx 139 (33.5%)
Janowak^30^	USA	Observational	2009–2013	168	–	–	CRT 93 (55.4%)
Kassis^31^	USA	Observational	2001–2011	7595	–	–	3478 (45.8%)
Kathiravetpillai^32^	The Netherlands	Observational	2001–2014	190	–	–	CRT 100%
Klevebro^33^	Sweden	Observational	2010–2017	2.332	–	–	–
Koeter^34^	The Netherlands	Observational	2009–2011	53	ACC 49 (92.5%), SCC 4 (7.5%)	–	CRT 100%
Koyanagi^35^	Japan	Observational	2014–2015	40	ACC 4 (10.0%), SCC 36 (90.0%)	Cervical, intrathoracic and GEJ	15 (30.0%)
Kruhilikava^36^	Denmark	Observational	2003–2010	285	–	–	–
Markar^37^	USA	Observational	1991–2011	500	–	–	–
McBee^38^	USA	Observational	2016–2018	1.260	ACC and SCC	Cervical, intrathoracic and GEJ	–
Miki^39^	Japan	Observational	2000–2015	158	–	Cervical, intrathoracic and GEJ	CTx 35 (22.2%)
Mitzman^40^	USA	Observational	2009–2016	9.389	–	–	–
Miyawaki^41^	Japan	Observational	2013–2017	188	ACC and SCC	–	–
Murphy^42^	USA	Observational	2002–2008	191	–	–	–
Okamura^43^	Japan	Observational	2011–2015	300	ACC and SCC	–	–
Rutegard^44^	Sweden	Observational	2001–2005	567	ACC 466 (82.2%), SCC 149 (26.7%)	Cervical, intrathoracic and GEJ	33 (5.8%)
Rutegard^45^	Sweden	Observational	2001–2005	559	ACC 449 (80.3%), SCC 110 (19.7%)	Cervical, intrathoracic and GEJ	29 (5.2%)
Saito^46^	Japan	Observational	2007–2015	90	ACC 3 (3.3%), SCC 87 (96.7%)	–	CTx 29 (32.2%)
Salem^47^	USA	Observational	2010–2013	129	–	–	–
Sato^48^	Japan	Observational	2013–2019	248	ACC 213 (85.9%), SCC 21 (8.5%)	–	–
Scarpa^49^	Italy	Observational	2008–2012	181	–	–	–
Schlottmann^50^	USA	Observational	2000–2014	5.243	–	–	–
Shichinohe^51^	Japan	Observational	2009–2012	483	–	–	–
Takeuchi^52^	Japan	Observational	2011	5.354	ACC and SCC	Cervical, intrathoracic and GEJ	1.005 (18.8%)
Werf^53^	The Netherlands	Observational	2011–2016	3.091	ACC and SCC	Intrathoracic and GEJ	CRT 3.091 (100%)
Zhao^54^	China	Observational	2010–2016	273	SCC 273 (100.0%)	–	0%

Author (year)	MI/open/both	Transthoracic/transhiatal	Location of anastomosis	Type of anastomosis	AL	Major complications*	30-Day mortality	Investigated prognostic factors
Alexiou^16^	100% open	–	Thoracic	–	29 (5.5%)	–	–	Age
Aoyama^17^	–	–	Cervical	–	44 (36.1%)	–	–	Age, sex, smoking, alcohol usage, tumor stage
Berkelmans^18^	100% MI	Both	–	–	15 (16.9%)	–	–	Sex, neoadjuvant therapy, ASA score, any comorbidity, cardiovascular comorbidity, pulmonary comorbidity, vascular comorbidity, diabetes, renal disease, steroid use, BMI
Borggreve^19^	Both	Both	–	Handsewn and stapled	104 (25.6%)	–	–	Sex, histology, neoadjuvant therapy, ASA score, cardiovascular comorbidity, pulmonary comorbidity, diabetes, smoking
Busweiler^20^	Both	Both	Cervical and thoracic	–	311 (12.4%)	–	59 (2.4%)	Age, sex, ASA score
Daele^21^	Both	Transthoracic	Thoracic	Stapled	12 (3.0%)	–	–	Age, sex, histology, neoadjuvant radiotherapy, neoadjuvant chemotherapy, ASA score, cardiovascular comorbidity, diabetes, renal disease, hypertension, previous surgery, smoking, preoperative weight loss
Filip^22^	Both	–	–	–	–	20 (12.0%)	–	Sex, tumor localization, histology, neoadjuvant therapy, ASA score, cardiovascular comorbidity, pulmonary comorbidity, vascular comorbidity, diabetes, renal disease, hepatic disease, HIV, preoperative weight loss
Fjederholt^23^	–	Both	–	–	42 (7.5%)	–	–	Sex, ASA score, Charlson index, smoking, tumor stage
Fogh^24^	–	–	–	–	32 (12.3%)	–	14 (5.4%)	Age, sex
Gao^25^	100% MI	Both	–	Handsewn and stapled	12 (12.5%)	–	–	Age, sex, neoadjuvant therapy, comorbidity, pulmonary comorbidity, diabetes, hypertension, alcohol
Goense^26^	100% MI	Both	–	Handsewn and stapled	40 (24.0%)	–	–	Sex, neoadjuvant therapy, ASA score, cardiovascular comorbidity, pulmonary comorbidity, vascular comorbidity, diabetes, renal disease, hypertension, smoking
Gooszen^27^	Both	–	Cervical and thoracic		656 (19.6%)	–	–	Sex, tumor localization, histology, neoadjuvant therapy, ASA score, cardiovascular comorbidity, pulmonary comorbidity, vascular comorbidity, diabetes, neurological comorbidity, hypertension, previous surgery, tumor stage
Hall^28^	Both	–	–	–	127 (13.9%)	–	–	Sex, histology, neoadjuvant radiotherapy, ASA score, ADL dependency, cardiovascular comorbidity, pulmonary comorbidity, bleeding disorder, diabetes, renal disease, steroid use, smoking, preoperative weight loss, tumor stage
Harustiak^29^	–	Both	Thoracic	Handsewn and stapled	56 (13.5%)	–	–	Sex, neoadjuvant therapy, neoadjuvant therapy, diabetes, hypertension, BMI
Janowak^30^	Both	Both	–	–	–	58 (35.0%)	–	Age, sex, neoadjuvant therapy, ASA score, cardiovascular comorbidity, pulmonary comorbidity, diabetes, renal disease, smoking, BMI
Kassis^31^	Both	Both	–	–	804 (10.6%)	–	–	Sex, neoadjuvant therapy, ASA score, cardiovascular comorbidity, vascular comorbidity, diabetes, renal disease, previous surgery, hypertension, steroid use, history of malignancy, smoking, BMI
Kathiravetpillai^32^	Both	–	–	–	50 (26.3%)	39 (20.5%)	9 (4.7%)	Interval neoadjuvant and surgery
Klevebro^33^	Both	–	–	–	312 (13.3%)	1383 (59.3%)	42 (1.8%)	Cardiovascular comorbidity, pulmonary comorbidity
Koeter^34^	Both	–	Cervical	Handsewn and stapled	13 (24.5%)	–	–	Sex, histology, ASA score, comorbidity
Koyanagi^35^	Both	Both	Cervical	Handsewn and stapled	7 (17.5%)	–	–	Sex, tumor localization, histology, neoadjuvant therapy, smoking, tumor stage
Kruhilikava^36^	–	–	–	–	24 (8.4%)	62 (21.8%)	7 (2.5%)	BMI
Markar^37^	–	–	–	–	18 (3.6%)	–	3 (0.6%)	Age
McBee^38^	Both	–	–	–	171 (13.6%)	–	34 (2.7%)	BMI ≥ 30 kg/m^2^
Miki^39^	100% MI	–	–	–	–	30 (23.4%)	–	Age, sex, tumor localization, neoadjuvant therapy, pulmonary comorbidity, diabetes, BMI < 25 kg/m^2^
Mitzman^40^	Both	–	–	–	–	–	321 (3.4%)	BMI
Miyawaki^41^	–	Transthoracic	Cervical	Handsewn	29 (15.4%)	–	–	Sex, neoadjuvant therapy pulmonary comorbidity, diabetes, hypertension, tumor stage
Murphy^42^	Both	–	–	–	16 (8.4%)	–	–	Comorbidity, smoking, alcohol, tumor stage
Okamura^43^	Both	–	Cervical	Handsewn and stapled	35 (11.7%)	–	–	Age, sex, histology, neoadjuvant therapy, cardiovascular comorbidity, pulmonary comorbidity, diabetes, neurological comorbidity, hepatic disease, hypertension, smoking, HbA1c
Rutegard^44^	–	Both	Thoracic	–	–	154 (25.0%)	–	Sex, histology, neoadjuvant therapy, any comorbidity, tumor stage
Rutegard^45^	–	Both	Thoracic	Handsewn and stapled	44 (7.9%)	–	–	Sex, histology, neoadjuvant therapy, any comorbidity, tumor stage
Saito^46^	100% MI	Both	–	–	–	32 (35.6%)	–	Sex, histology, neoadjuvant therapy, ASA score, cardiovascular comorbidity, diabetes, smoking, alcohol, BMI, tumor stage
Salem^47^	100% MI	–	–	–	5 (3.9%)	–	–	BMI
Sato^48^	Both	–	Thoracic	–	38 (15.3%)	–	–	Sex, histology, neoadjuvant therapy, pulmonary comorbidity, diabetes, hypertension, tumor stage
Scarpa^49^	Both	Both	–	–	8 (4.4%)	20 (11.0%)	2 (1.1%)	Age
Schlottmann^50^	–	–	–	–	297 (5.7 %)	–	–	Age
Shichinohe^51^	–	–	–	–	54 (11.1%)	132 (27.3%)	–	Sex, malnutrition
Takeuchi^52^	Both	Both	Both	–	–	–	244 (4.6%)	Sex, neoadjuvant therapy, ASA score, renal disease
Werf^53^	Both	Both	Cervical and thoracic	Hand sewn and stapled	341 (11.0%)	185 (6.0%)	106 (3.4%)	Interval neoadjuvant and surgery
Zhao^54^	100% MI	–	–		19 (7.0%)	25 (9.2%)	0 (0.0%)	Age

*ASA* American Society of Anesthesiologists, *AL* anastomotic leakage, *BMI* body mass index, *GEJ* gastroesophageal junction, *MI* minimally invasive, *ACC* adenocarcinoma, *CRT* chemoradiotherapy, *CTx* chemotherapy, *RT* radiotherapy, *SCC* squamous cell carcinoma

^*^Major complications defined as Clavien–Dindo ≥ IIIa

### Anastomotic Leakage

A total of 37 prognostic factors for anastomotic leakage were described in 31 studies; all were included in the meta-analyses (Table 2). Ten factors were significantly associated with anastomotic leakage, and one protective factor was identified.

**Table 2:**
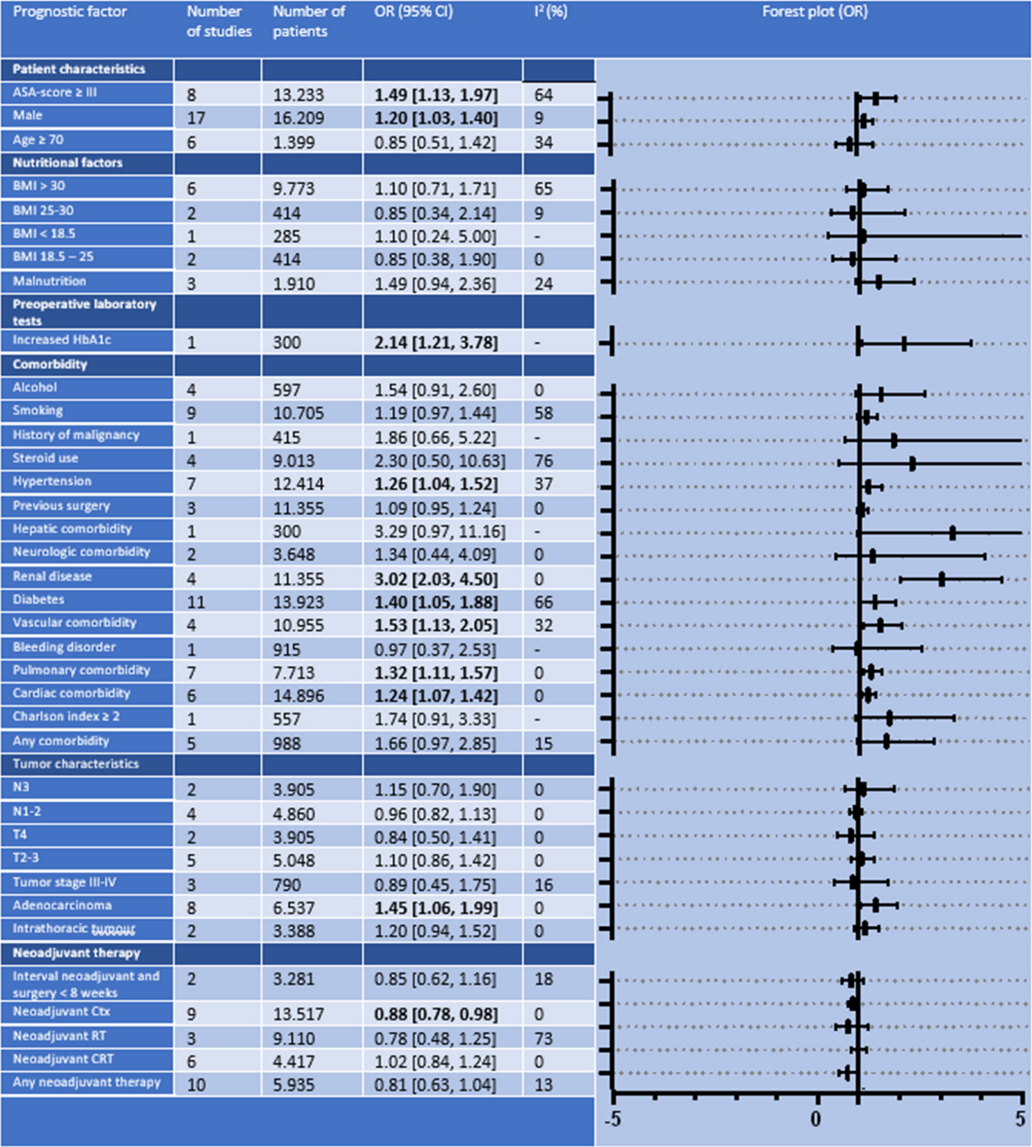
Results of meta-analyses identifying patient-related prognostic factors for anastomotic leakage

*ASA* American Society of Anesthesiologists, *BMI* body mass index, *CRT* chemoradiotherapy, *Ctx* chemotherapy, *OR* odds ratio, *RT* radiotherapy

Renal disease was the most prominent prognostic factor for anastomotic leakage with an OR of 3.02 (95% CI 2.03–4.50; *P * < 0.01). In addition, vascular comorbidity (OR 1.53; 95% CI 1.13–2.05; *P *< 0.01), diabetes (OR 1.40; 95% CI 1.05–1.88; *P *< 0.01), pulmonary comorbidity (OR 1.32; 95% CI 1.11–1.57; *P *< 0.01), hypertension (OR 1.26; 95% CI 1.04–1.52; *P *= 0.02), and cardiac comorbidity (OR 1.24; 95% CI 1.07–1.42; *P < *0.01) were significantly associated with anastomotic leakage. ASA score ≥ III also significantly increased the risk of anastomotic leakage (OR 1.49; 95% CI 1.13–1.97; *P *= 0.04). Males were at greater risk for anastomotic leakage than females (OR 1.20; 95% CI 1.03–1.40; *P *= 0.02). Anastomotic leakage occurred more often after surgery for adenocarcinoma compared with squamous cell carcinoma (OR 1.45; 95% CI 1.06–1.99; *P *= 0.02).

Increased hemoglobin A1c (HbA1c) was also associated with anastomotic leakage (OR 2.14; 95% CI 1.21–3.78; *P *< 0.01) but was only described by one study.^43^ Therefore, meta-analysis was not possible.

Patients receiving neoadjuvant chemotherapy were at lower risk for anastomotic leakage (OR 0.88; 95% CI 0.78–0.98; *P *= 0.04).

An analysis of studies only including minimally invasive esophagectomy showed no significant associations (Supplementary File 4).

### Major Complications

A total of 23 prognostic factors for major postoperative complications (CD ≥ IIIa) were described in 11 studies and were used in the meta-analyses (Table 3). Of these factors, four were significantly associated with major complications, of which male sex was the most prominent (OR 4.50; 95% CI 1.21–16.64; *P *= 0.02). In addition, cardiac comorbidity (OR 1.53; 95% CI 1.25–1.87; *P *< 0.01) and diabetes (OR 1.93; 95% CI 1.14–3.26; *P *= 0.01) were significantly associated with major complications. The presence of any comorbidity was also associated with major complications but was described in only one study (OR 1.69; 95% CI 1.12–2.55; *P *= 0.01). A time interval between neoadjuvant therapy and surgery of < 8 weeks was associated with fewer major complications (OR 0.68; 95% CI 0.50–0.93; *P *= 0.01).

**Table 3:**
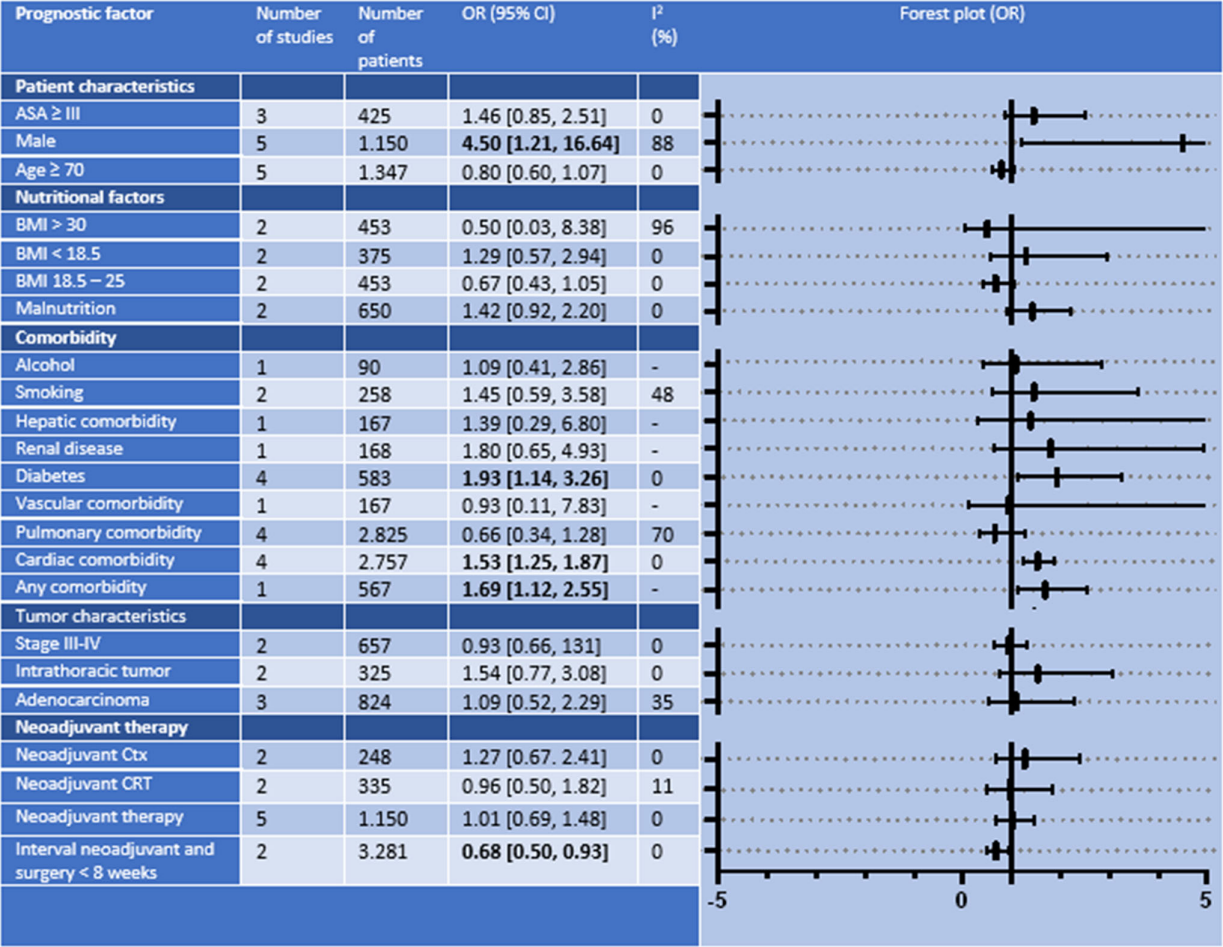
Results of meta-analyses identifying patient-related prognostic factors for major complications (Clavien–Dindo ≥ IIIa)

*ASA* American Society of Anesthesiologists, *BMI* body mass index, *Ctx* chemotherapy, *CRT* chemoradiotherapy, *OR* odds ratio

### Mortality

Fifteen prognostic factors for 30-day/in-hospital mortality were identified in 12 studies and used for meta-analyses (Table 4). Of these factors, six were significantly associated with increased mortality rates. ASA ≥ III (OR 2.77; 95% CI 1.80–4.26; *P < *0.01), cardiac comorbidity (OR 2.40; 95% CI 1.72–3.35; *P < *0.01), age 70 years or older (OR 2.06; 95% CI 1.66–2.56; *P *< 0.01), and BMI of 18.5–25 kg/m^2^ (OR 1.41; 95% CI 1.11–1.78; *P < *0.01) were significantly associated with higher risk of mortality. In addition, habitual alcohol usage (OR 3.10; 95% CI 2.26–4.25; *P < *0.01) and renal disease (OR 2.85; 95% CI 1.71–4.74; *P < *0.01) were significantly associated with increased mortality rates but were described in only one study. Overweight (BMI 25–30 kg/m^2^) (OR 0.40; 95% CI 0.30–0.53; *P < *0.01) and an interval between neoadjuvant therapy and surgery of < 8 weeks (OR 0.54; 95% CI 0.35–0.85; *P < *0.01) were associated with lower mortality rates.

**Table 4:**
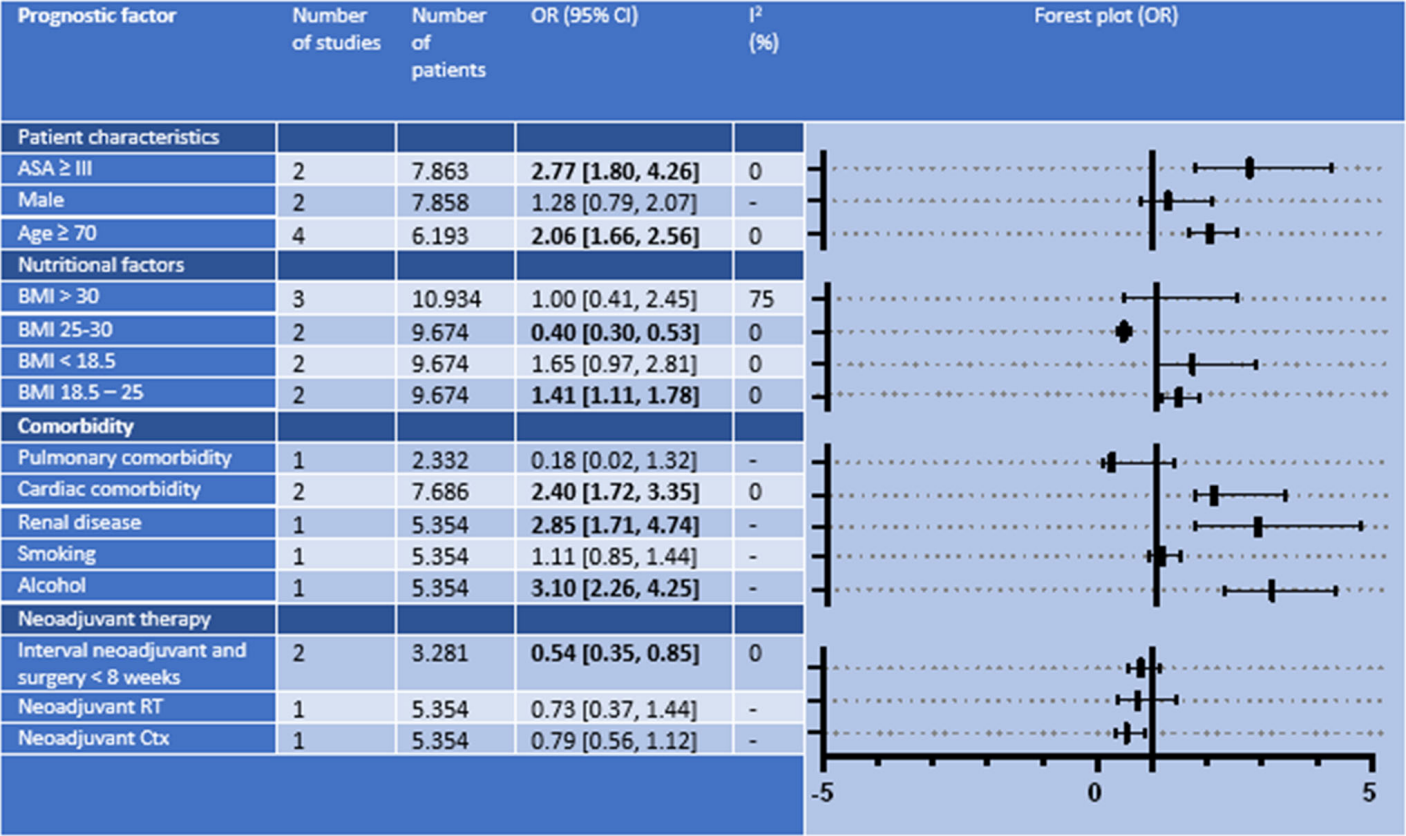
Results of meta-analyses identifying patient-related prognostic factors for 30-day/in-hospital mortality

*ASA* American Society of Anesthesiologists, *BMI* body mass index, *Ctx* chemotherapy, *OR* odds ratio, *RT* radiotherapy

## Discussion

This is the first study to present a systematic review and meta-analyses describing prognostic factors for anastomotic leakage, major complications, and 30-day/in-hospital mortality following esophageal cancer surgery. Thirty-nine studies were included, providing a comprehensive and quantitative overview of the available literature. After analyses of 37 potential prognostic factors described in literature, renal disease, vascular comorbidity, diabetes, pulmonary, hypertension, cardiac comorbidity, ASA score ≥ III, male sex, and adenocarcinoma tumor histology were prognostic factors for anastomotic leakage. Patients receiving neoadjuvant chemotherapy had a lower risk for anastomotic leakage. Male sex, cardiac comorbidity, and diabetes were prognostic factors for major complications. In the current study, age > 70 years, ASA score ≥ III, cardiac comorbidity, and BMI of 18.5–20 kg/m^2^ were prognostic factors for mortality whereas BMI of 25–30 kg/m^2^ appeared preventive of mortality. A time interval of < 8 weeks between neoadjuvant therapy and surgery was associated with lower major complication and mortality rates.

### Patient Characteristics

Although the observed associations were heterogeneous, this study shows that male sex was associated with both higher anastomotic leakage and major complication rates. This might be a result of the higher incidence of smoking and alcohol consumption in the male population.^55^ Another theory described in literature is that cortisol-induced sex hormones vary among men and women, making males more susceptible to postoperative complications after surgically induced stress.^56^ In the current study, older patients are at higher risk for postoperative mortality than younger patients; age did not seem to impact anastomotic leakage and major complication rates. This might be caused by an increased susceptibility for failure to rescue in the elderly.^5^ In the elderly, decreased preoperative performance status as demonstrated by a higher ASA score and/or comorbidities, such as cardiac and pulmonary comorbidity, might result in worse short-term outcomes.^33^

### Comorbidity

As shown in this study, comorbidity is a prognostic factor for the occurrence of postoperative complications. The presence of comorbidities might, besides poorer physical performance, also implicate a greater presence of artery calcifications, which was shown by Goense et al. to be independently associated with anastomotic leakage.^57^ Additionally, the association between diabetes and major complications is well understood, as hyperglycemia induces microvascular damage that subsequently reduces healing capacity.^58^

### Body Mass Index

This study showed that BMI of 18.5–20 kg/m^2^ is associated with postoperative mortality. Patients with BMI between 25 and 30 kg/m^2^, however, tended to have lower risk for mortality. Previous studies have shown that preoperative weight loss and a lower BMI make patients more susceptible for failure to rescue.^4,5^ Patients with higher BMI at baseline might have more physical reserves (i.e., be less prone for catabolism), which prevents short-term adverse events. An even higher BMI (> 30 kg/m^2^) was not protective for mortality. This might be caused by the difficulty of surgery in the obese due to the high amount of visceral fat compromising intraoperative visibility and making the surgery more challenging.^59^

### Neoadjuvant Therapy

The current study also shows lower leakage rates after administration of neoadjuvant therapy. As the administration of neoadjuvant therapy is the standard of care for esophageal cancer, it may only be omitted in frail patients unable to withstand systemic therapy. This might explain the lower anastomotic leakage rates in patients undergoing neoadjuvant therapy compared with patients not receiving preoperative systemic therapy. Another prognostic factor observed in this study was the interval between neoadjuvant therapy and surgery. This study shows that an interval of > 8 weeks is associated with increased major complication and mortality rates. The higher rate of adverse events in patients with a prolonged interval may be subjected to selection bias. Van de Werf et al. showed that more frail patients had a longer interval.^53^ In these frail patients, the interval might have been used for preoperative optimization. Another explanation may be that the interval is prolonged due to toxicity and/or slower recovery from neoadjuvant therapy. However, especially in patients undergoing chemoradiotherapy, the longer interval might also complicate surgery because of increased postradiation scarring with increasing interval lengths.

### Tumor Histology

As shown in this study, adenocarcinoma tumor histology is a prognostic factor for anastomotic leakage after esophagectomy. A theory is that, based on the differences in pathogenesis of adenocarcinoma and squamous carcinoma, patient characteristics are different. For instance, adenocarcinoma is more common in overweight and obese patients, and in patients with more alcohol usage both are risk factors for anastomotic leakage.^36^ However, squamous cell carcinoma is more common in patients with habitual alcohol usage and smoking.^60^ Another difference between adenocarcinoma and squamous cell carcinoma is the localization, since adenocarcinoma is typically located more proximally. This localization is more suitable for cervical anastomosis, which is associated with a higher frequency of anastomotic leakage.^27^

### Surgical Techniques

Given the differences in incidence and severity of anastomotic leakage of cervical versus intrathoracic anastomosis, the risk factors for anastomotic leakage might also differ based on anastomotic location.^27^ Additionally, minimally invasive surgery is being used more in daily practice, but unfortunately many studies do not report open and minimally invasive procedures separately. Therefore, this meta-analysis was unable to make distinctions between different surgical techniques (e.g., location of anastomosis, minimally invasive surgery), since the included studies did not allow for stratified analyses.

### Perioperative Care

The identification of prognostic factors for adverse events after esophagectomy may provide opportunities to optimize perioperative care by treating or optimizing these prognostic factors preoperatively and thereby decreasing surgical risk. Reduction of postoperative morbidity and mortality may in turn reduce healthcare costs.^61^ Therefore, reduction of postoperative morbidity impacts healthcare at patient, hospital, and national levels. The prognostic factors described in the current study may contribute to focused and personalized preoperative care by enrolling patients with certain prognostic factors into (tailormade) prehabilitation programs. Currently, more generalized perioperative care programs are being studied and implemented in the form of ERAS protocols.^62^ As part of the ERAS protocols, lifestyle interventions (e.g., alcohol cessation) are introduced in daily practice.^63–65^ In addition, there is more focus on preoperative malnutrition and impaired physical capacity, which are shown to be negative prognostic factors for postoperative complications in this meta-analysis.^66^ Intra- and postoperative care are also being standardized in ERAS protocols (e.g., fluid therapy, opioid-sparing analgesia).^67^

The reduction of postoperative complications is important because complications are associated with reduced overall survival. Additionally, the reduction of complications positively impacts (progression-free) survival.^68^ It is thought that infectious complications lead to release of proinflammatory cytokines, which are related to tumor progression and metastasis.^69^ One might even argue that resection could be reconsidered in patients with multiple prognostic factors as definitive chemoradiotherapy might be a more well suited curative treatment option for such patients.^70,71^ However, one should keep in mind the reduced survival after definitive chemoradiotherapy.

With the use of neoadjuvant therapy, a window for preoperative optimization is opened. A systematic review showed that (p)rehabilitation programs for esophageal cancer patients can improve objective measures of physical fitness. However, effects on postoperative outcomes were less eminent.^72^ Nonetheless, preoperative exercise programs have been shown to significantly impact health-related quality of life.^73^ Several studies report that well-designed randomized controlled trials on prehabilitation programs are needed in order to prove their beneficial effects on short-term postoperative outcomes.^72,74^ They should focus on optimizable preoperative prognostic factors (e.g., malnutrition or vitamin deficiencies). Esophageal cancer patients are at high risk for malnutrition due to the anatomical localization of the tumor. Therefore, nutritional interventions are important in preoperative prehabilitation.^75^ This is supported by the results of the current study showing that patients with low BMI have increased risk of postoperative mortality. Slightly overweight patients even had reduced mortality rates. These results indicate that malnourishment and depletion of essential food substances are an important and modifiable prognostic factor in esophageal cancer surgery.

Identification of high-risk patients may indicate that changes in postoperative care are needed, for example, closer postoperative surveillance or delayed enteral feeding in high-risk patients. Closer postoperative surveillance might for instance be done by using wearable devices for continuous postoperative monitoring, even on the regular hospital ward. This might lead to more timely recognition and identification of postoperative adverse events, subsequently leading to earlier treatment and lower failure-to-rescue rates.^76,77^

The identified prognostic factors for major adverse outcomes after surgery are vital in clinical auditing. Comparing hospitals and providing clinicians with benchmarked outcome information is an important quality improvement tool.^78^ For fair hospital comparison, benchmarked information should be corrected for differences in case mix among hospitals. The current study provides prognostic factors for three major adverse events after esophagectomy that should be used for case-mix correction in clinical audits such as the Dutch Upper Gastrointestinal Cancer Audit (DUCA).^79^

### Limitations

This study had some limitations. Firstly, it provided an overview of multiple studies, creating a heterogeneous patient population. Additionally, definitions of prognostic factors (e.g., renal disease, cardiac comorbidity) used in literature are heterogeneous, making interpretation difficult. In addition, neoadjuvant therapy is currently standard of care, but this is not yet incorporated in all studies, compromising the external validity of the current study. As discussed, ERAS protocols influence postoperative outcomes, which may interfere with the results of this meta-analysis. However, none of the included studies reported on the use of ERAS protocols. The observational study design used in all the included studies may have hindered adequate interpretation of results. Additionally, most of the included studies were retrospective. Therefore, the current study is subjected to bias. However, it is suspected that, due to the high number of studies and patients included, this bias was limited. In current prognostic factor research, several limitations are known, such as publication bias, reporting bias, poor statistical analysis, and inadequate replication of findings.^80^ These meta-analyses used pooled data to calculate univariable ORs, which do not correct for potential confounding factors. Additionally, this study focuses on preoperative prognostic factors, whereas surgical factors, such as the type of anastomosis or surgery, may also contribute to the risk of postoperative major complications. Lastly, continuous variables such as BMI and age are reported as categorical variables, which is subjected to bias and may make risk estimates less useful.^81^

Future research should be directed towards prospective studies with well-documented prognostic factors, in addition to well-designed randomized controlled trials investigating the impact of preoperative prehabilitation programs for modifiable prognostic factors on surgical outcomes and quality of life. This should pave the way to enhanced personalized perioperative care.

### Conclusion

In conclusion, this systematic review and meta-analyses identified 37 prognostic factors that are associated with adverse events after esophageal cancer surgery. Cardiac comorbidity was identified as a prognostic factor for all three studied adverse outcomes (anastomotic leakage, major complications, and mortality). Male sex and diabetes were identified as prognostic factors for anastomotic leakage and major complications. ASA score > III and renal disease were shown to be associated with anastomotic leakage and mortality. Pulmonary comorbidity, vascular comorbidity, hypertension, and adenocarcinoma were prognostic factors for anastomotic leakage. Older age (> 70 years), habitual alcohol usage, and intermediate BMI were associated with increased risk for mortality. These factors should be used in case-mix correction models in national clinical audits. In addition, they also enable further research for accurate preoperative patient selection and personalized perioperative care ultimately aiming to reduce surgical morbidity and improve postoperative quality of life.

## Supplementary Information

Below is the link to the electronic supplementary material.

Supplementary file1 (DOCX 20 KB)

Supplementary file2 (DOCX 16 KB)

Supplementary file3 (DOCX 38 KB)

Supplementary file4 (DOCX 64 KB)
