# Supplementary material for: Patient-Related Prognostic Factors for Anastomotic Leakage, Major Complications, and Short-Term Mortality Following Esophagectomy for Cancer: A Systematic Review and Meta-Analyses
Source: Ann Surg Oncol. 2021 Sep 5;29(2):1358–73. doi: 10.1245/s10434-021-10734-3 (PMC8724192; doi:10.1245/s10434-021-10734-3)
Supplement: Supplementary file 2 — Supplementary file2 (DOCX 16 KB) [file 10434_2021_10734_MOESM2_ESM.docx]

# **SUPPLEMENTARY FILE 2 – Search Strategy**

**Pubmed search**

("Esophagectomy"[ti] OR "Esophagectomies"[ti] OR "Oesophagectomy"[ti] OR "Oesophagectomies"[ti] OR (("Esophagus"[Majr] OR "Esophageal Neoplasms"[Majr] OR "Esophagus"[ti] OR "Oesophagus"[ti] OR "Esophagi"[ti] OR "Oesophagi"[ti] OR "Esophageal"[ti] OR "Oesophageal"[ti] OR "Esophaguses"[ti] OR "Oesophaguses"[ti]) AND ("Surgical Procedures, Operative"[Majr:NoExp] OR "surgery"[ti] OR "surgeries"[ti] OR "surgical"[ti] OR "resection"[ti] OR "resections"[ti]))) AND ("Risk Factors"[Mesh] OR "Risk Factor"[tw] OR "Risk Factors"[tw] OR "determinant"[tw] OR "determinants"[tw] OR "Comorbidity"[Mesh] OR "Comorbidity"[tw] OR "Comorbidities"[tw] OR "multimorbidity"[tw] OR "multimorbidities"[tw] OR "Age Factors"[Mesh] OR "Age"[tw] OR "Body Weight"[Mesh:NoExp] OR "Body Weight"[tw] OR "Body Weights"[tw] OR "Thinness"[Mesh] OR "thinness"[tw] OR "Underweight"[tw] OR "Overweight"[Mesh] OR "obesity"[tw] OR "obese"[tw] OR "Overweight"[tw] OR "Body Mass Index"[Mesh] OR "Body Mass Index"[tw] OR "BMI"[ti] OR "Sarcopenia"[Mesh] OR "Sarcopenia"[tw] OR "sarcopenic"[tw] OR "Smoking"[Mesh] OR "Smoking Cessation"[Mesh] OR "Tobacco Use"[Mesh] OR "smoking"[tw]) AND ("Postoperative Complications"[majr] OR "Postoperative Hemorrhage"[majr] OR (("Postoperative Period"[Majr:NoExp] OR "postoperative"[ti] OR "post-operative"[ti]) AND ("adverse"[ti] OR "hemorrhage"[ti] OR "hemorrhages"[ti] OR "haemorrhage"[ti] OR "haemorrhages"[ti] OR "complication"[ti] OR "complications"[ti] OR "hemoperitoneum"[ti] OR "Anastomotic Leak"[majr] OR "Anastomotic Leak"[ti] OR "Anastomotic Leaks"[ti] OR "anastomotic leakage"[ti] OR "anastomotic leakages"[ti] OR "Shock, Surgical"[majr] OR "surgical shock"[ti] OR "Heart Arrest"[majr] OR "Heart Arrest"[ti] OR "cardiac arrest"[ti] OR "Brain Ischemia"[majr] OR "Brain Ischemia"[ti] OR "Brain Ischemias"[ti] OR "Brain Ischaemia"[ti] OR "Brain Ischaemias"[ti] OR "brain infarction"[ti] OR "brain infarctions"[ti] OR "Myocardial Infarction"[majr] OR "Myocardial Infarction"[ti] OR "Myocardial Infarctions"[ti] OR "Stroke"[majr] OR "Stroke"[ti] OR "Strokes"[ti] OR "CVA"[ti] OR "cerebrovascular accident"[ti] OR "cerebrovascular accidents"[ti] OR "Pulmonary Embolism"[majr] OR "Pulmonary Embolism"[ti] OR "Pulmonary Embolisms"[ti] OR "pulmonary thromboembolism"[ti] OR "pulmonary thromboembolisms"[ti] OR "Sepsis"[majr] OR "Sepsis"[majr] OR "sepses"[ti] OR "septic"[ti] OR "Multiple Organ Failure"[majr] OR "Multiple Organ Failure"[ti]))) NOT ("animals"[mesh] NOT "humans"[mesh]) NOT (("infant"[mesh] OR "child"[mesh] OR "adolescent"[mesh]) NOT "adult"[mesh])

**Embase search**

("Esophagectomy".ti. OR "Esophagectomies".ti. OR "Oesophagectomy".ti. OR "Oesophagectomies".ti. OR ((*esophagus/ OR *gastroesophageal junction/ OR *esophagus tumor/ or exp *esophagus cancer/ OR "Esophagus".ti. OR "Oesophagus".ti. OR "Esophagi".ti. OR "Oesophagi".ti. OR "Esophageal".ti. OR "Oesophageal".ti. OR "Esophaguses".ti. OR "Oesophaguses".ti.) AND (*surgery/ OR abdominal surgery/ OR exp *cancer surgery/ OR *general surgery/ OR *major surgery/ OR "surgery".ti. OR "surgeries".ti. OR "surgical".ti. OR "resection".ti. OR "resections".ti.))) AND (risk factor/ OR "Risk Factor".ti,ab. OR "Risk Factors".ti,ab. OR "determinant".ti,ab. OR "determinants".ti,ab. OR comorbidity/ OR "Comorbidity".ti,ab. OR "Comorbidities".ti,ab. OR "multimorbidity".ti,ab. OR "multimorbidities".ti,ab. OR age/ OR "Age".ti,ab. OR body weight/ OR "Body Weight".ti,ab. OR "Body Weights".ti,ab. OR underweight/ OR "thinness".ti,ab. OR "Underweight".ti,ab. OR exp obesity/ OR "obesity".ti,ab. OR "obese".ti,ab. OR "Overweight".ti,ab. OR body mass/ OR "Body Mass Index".ti,ab. OR "BMI".ti. OR sarcopenia/ OR "Sarcopenia".ti,ab. OR "sarcopenic".ti,ab. OR exp smoking/ OR smoking cessation/ OR exp "tobacco use"/ OR "smoking".ti,ab.) AND (exp *postoperative complication/ OR ((*postoperative period/ OR "postoperative".ti. OR "post-operative".ti.) AND ("adverse".ti. OR "hemorrhage".ti. OR "hemorrhages".ti. OR "haemorrhage".ti. OR "haemorrhages".ti. OR "complication".ti. OR "complications".ti. OR "hemoperitoneum".ti. OR "Anastomotic Leak".ti. OR "Anastomotic Leaks".ti. OR "anastomotic leakage".ti. OR "anastomotic leakages".ti. OR exp *shock/ OR "surgical shock".ti. OR exp *heart arrest/ OR "Heart Arrest".ti. OR "cardiac arrest".ti. OR exp *brain ischemia/ OR "Brain Ischemia".ti. OR "Brain Ischemias".ti. OR "Brain Ischaemia".ti. OR "Brain Ischaemias".ti. OR "brain infarction".ti. OR "brain infarctions".ti. OR exp *heart infarction/ OR "Myocardial Infarction".ti. OR "Myocardial Infarctions".ti. OR exp *cerebrovascular accident/ OR "Stroke".ti. OR "Strokes".ti. OR "CVA".ti. OR "cerebrovascular accident".ti. OR "cerebrovascular accidents".ti. OR exp *venous thromboembolism/ OR "Pulmonary Embolism".ti. OR "Pulmonary Embolisms".ti. OR "pulmonary thromboembolism".ti. OR "pulmonary thromboembolisms".ti. OR exp *sepsis/ OR "sepses".ti. OR "septic".ti. OR exp *multiple organ failure/ OR "Multiple Organ Failure".ti.))) NOT ("animal"/ NOT "human"/) NOT (exp juvenile/ NOT exp adult/) NOT "conference abstract".pt.
