# Supplementary material for: Patient-Related Prognostic Factors for Anastomotic Leakage, Major Complications, and Short-Term Mortality Following Esophagectomy for Cancer: A Systematic Review and Meta-Analyses
Source: Ann Surg Oncol. 2021 Sep 5;29(2):1358–73. doi: 10.1245/s10434-021-10734-3 (PMC8724192; doi:10.1245/s10434-021-10734-3)
Supplement: Supplementary file 3 — Supplementary file3 (DOCX 38 KB) [file 10434_2021_10734_MOESM3_ESM.docx]

# **SUPPLEMENTARY FILE 3 – Risk of Bias**


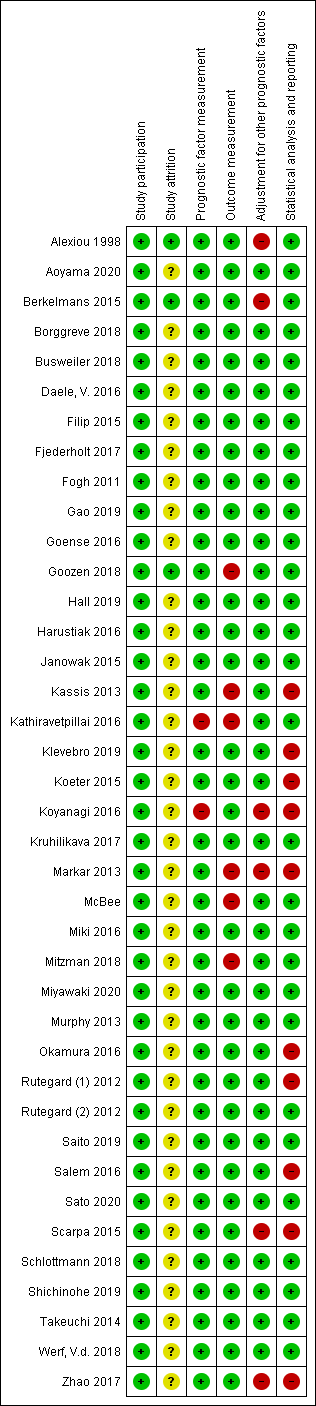


**Table S1 -** Table showing the classification of study quality of all included studies using the Quality In Prognosis Studies (QUIPS) tool ^1^. On the horizontal axis show the different assed domains per study. The vertical axis shows all included studies. The green bullet = low risk of bias, yellow = unclear risk of bias, red = high risk of bias.


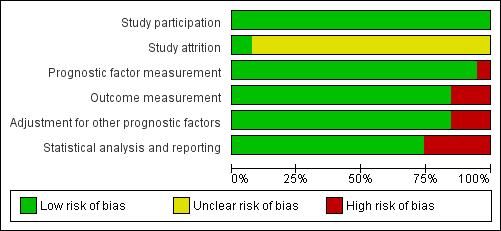


**Table S2 –** Summary table showing an overview of classification of study quality of all included studies using the QUIPS tool as shown in table S1.

**REFERENCES**

1. Hayden, J.A., et al., *Assessing bias in studies of prognostic factors.* Ann Intern Med, 2013. **158**(4): p. 280-6.
