# Supplementary material for: Patient-Related Prognostic Factors for Anastomotic Leakage, Major Complications, and Short-Term Mortality Following Esophagectomy for Cancer: A Systematic Review and Meta-Analyses
Source: Ann Surg Oncol. 2021 Sep 5;29(2):1358–73. doi: 10.1245/s10434-021-10734-3 (PMC8724192; doi:10.1245/s10434-021-10734-3)
Supplement: Supplementary file 4 — Supplementary file4 (DOCX 64 KB) [file 10434_2021_10734_MOESM4_ESM.docx]

# **SUPPLEMENTARY FILE 4 – Minimally Invasive Esophagectomy**


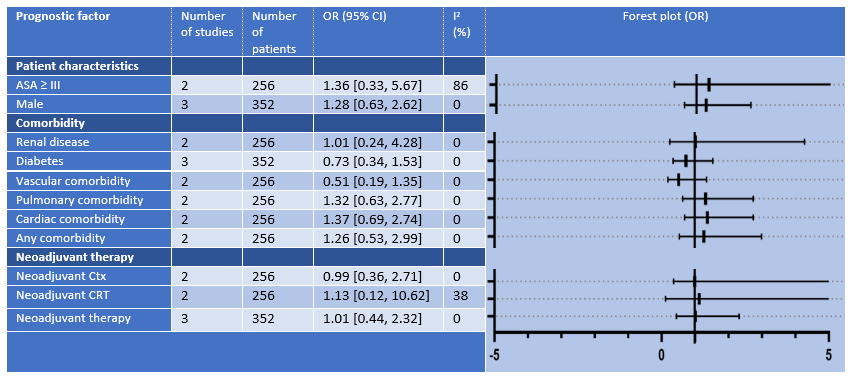


**Table S3 -** Results of meta-analyses identifying patient-related prognostic factors for anastomotic leakage after minimally invasive esophagectomy. ASA-score; American Society of Anesthesiologists score, CRT; Chemoradiotherapy, Ctx; Chemotherapy, OR; Odds Ratio.
